# Dendritic Cell Subsets in Oral Mucosa of Allergic and Healthy Subjects

**DOI:** 10.1371/journal.pone.0154409

**Published:** 2016-05-11

**Authors:** Susanne M. Reinartz, Joost van Tongeren, Danielle van Egmond, Esther J. J. de Groot, Wytske J. Fokkens, Cornelis M. van Drunen

**Affiliations:** Department of Otorhinolaryngology, Academic Medical Center, Amsterdam, the Netherlands; French National Centre for Scientific Research, FRANCE

## Abstract

Immunohistochemistry was used to identify, enumerate, and describe the tissue distribution of Langerhans type (CD1a and CD207), myeloid (CD1c and CD141), and plasmacytoid (CD303 and CD304) dendritic cell subsets in oral mucosa of allergic and non-allergic individuals. Allergic individuals have more CD141+ myeloid cells in epithelium and more CD1a+ Langerhans cells in the lamina propria compared to healthy controls, but similar numbers for the other DC subtypes. Our data are the first to describe the presence of CD303+ plasmacytoid DCs in human oral mucosa and a dense intraepithelial network of CD141+ DCs. The number of Langerhans type DCs (CD1a and CD207) and myeloid DCs (CD1c), was higher in the oral mucosa than in the nasal mucosa of the same individual independent of the atopic status.

## Introduction

Antigen-presenting dendritic cells (DCs) form a heterogeneous group of cells based on what cellular lineages they originate from and their stimulatory or suppressive contribution to immune responses. The DC population of upper and lower airway mucosa and of peripheral blood have been shown to comprise of Langerhans type, myeloid, and plasmacytoid DCs using immunohistochemical staining with specific cell markers [1–4]. The goal of our study was to identify and characterize dendritic cell subtypes in human oral mucosal biopsies by immunohistochemical staining in allergic and non-allergic individuals. Furthermore, we performed a detailed comparison of oral and nasal mucosal DCs in the same individuals.

The interplay between the immune stimulatory or suppressive activities of DCs is important for both the induction of an immune response and the maintenance of local homeostasis. In respiratory mucosa, myeloid DCs (mDCs) play an essential role in sustaining a chronic eosinophilic airway inflammation [5], whereas plasmacytoid DCs (pDC) are important in maintaining tolerance to inhaled harmless antigen [6]. Previously we have shown that the mDC/pDC ratio in nasal mucosa is similar for allergic and healthy subjects at baseline, but that after nasal allergen provocation this ratio in healthy subjects decreases while in allergic subjects this ratio remains unchanged. This not only suggested the induction of an immunosuppressive activity in healthy individuals upon allergen encounter, but even more importantly, that allergic individuals seem to lack this immunosuppressive activity.

For human oral mucosa, the interplay between different subtypes of DCs in relation to immune stimulatory or suppressive activities is less clear. This is due to only a few studies addressing these issues. In regards to the composition of DC subtypes in oral mucosa in mice three distinct subtypes of oral DCs were characterized: CD207+ Langerhans cells, CD11b+CD11c+/- myeloid DCs, and B220+120G8+ plasmacytoid DCs [7]. The CD 207+ DCs may also express CD103, which is identified as the main migratory subtype able to cross-present viral and self-antigens, critical for the initiation of CD8+ T cell responses [8]. In human oral mucosa also Langerhans type cells and myeloid DCs have been detected, with the Langerhans cells representing the predominating DC population [9]. Contrary to the findings in mice, plasmacytoid DCs characterized by CD123+ could not be detected in the human oral mucosa of atopic and non-atopic individuals [10–12]. In addition to differences in DC composition, also functional aspects of oral DC are potentially different to DC at other mucosal surfaces. Oral antigen-presenting cells have been considered to exhibit a partly tolerogenic phenotype, leading to suppression of local and systemic immune responses after orally administering antigens. This concept is also referred to as oral tolerance, and can be seen as a form of peripheral tolerance that could prevent harmful responses to harmless antigens, such as antigens from food or commensal bacteria. In mice studies some of these differences with respiratory DCs emerge where CD11b+CD11c- DCs only induced IFN-gamma production by T-cells, while oral CD11b+CD11c+ and B220+120G8+ DCs induced IFN-gamma and IL-10 secreting T cells. Given that in none of these experiments any expression of the cytokine IL-5 could be detected, it would seem that the immune system of the oral mucosa is more attuned to Th1 response (INF-gamma) and immune suppressive responses (IL-10) than to Th2 responses (IL-5). Indeed also in human studies, the presence of immunosuppressive enhancing molecules (B7-H2 and B7-H3) on oral Langerhans cells [7] and relative high mRNA levels of IL-10 and TGF-beta [9,10] suggest such an immune suppressive environment. However, it is not clear how the immune system deals with the multitude of antigenic triggers from the environment, not only making sure that the response is appropriately Th1 or Th2, but even making sure that such a response is at all required. It may well be that the suggested lack of Th2 responses in oral mucosa could be related to or play a role in the effectiveness of sublingual immunotherapy (SLIT). Indeed, Langerhans type cells in human oral mucosa are thought to contribute to the effectiveness of SLIT [9,10,13–15].

In order to get a better understanding of the local oral mucosal immune system, we have quantified the oral Langerhans type cells as well as oral myeloid and plasmacytoid DCs and studied their local tissue distribution. The subject population we have investigated in this manuscript is the same population where we have previously reported the dynamics of the local nasal mucosal DC populations [2]. This also allows us to explore the relationship of oral DCs with the allergic state and the direct comparison of nasal and oral DC populations in single individuals.

## Methods

### Subjects

The subjects in this study were adults with allergic rhinitis (AR) with or without asthma (n = 52) of which 14 subjects with seasonal AR symptoms and grass pollen mono-sensitization, 9 subjects with perennial rhinitis and house dust mite mono-sensitization, and 29 subjects with perennial rhinitis with increasing symptoms during the pollen season and poly-sensitization, including house dust mite and grass pollen. The diagnosis of AR was based on a history of rhinitis symptoms for at least two years and a positive skin prick test. At screening, all subjects were tested for a panel of 10 common inhalant allergens (ALK-Abello BV, Nieuwegein, Netherlands). A skin prick test was considered positive when the wheal diameter was 3 mm larger than that produced by the negative control after 15 minutes. These allergic subjects were compared with healthy controls (n = 14) without any rhinitis symptoms and a negative skin prick test. All subjects were non-smokers and were clinically stable. Subjects were excluded if they suffered from a disorder likely to interfere with the test results. All medication for AR treatment was appropriately stopped before enrolment. Subjects with asthma were allowed to participate in the study if they were able to stop their asthma medication during the study. Thus, 66 eligible subjects (52 patients and 14 controls; 41 female, median age 23.8 years; range 18–62 years) were included in this study. Demographic characteristics were comparable between groups. All participants gave written informed consent to the study, which was approved by the medical ethics committee of the Academic Medical Center, Amsterdam, Netherlands under project number MEC03/201.

### Study Design

Oral and nasal mucosal biopsies were taken from the same individuals at the same time point, and were stained with several DC-markers to identify and enumerate different dendritic cell subsets. We compared the number of DCs per subset in the oral mucosa between allergic subjects and healthy controls, and compared the number of DCs per subset between nasal and oral mucosa.

### Oral and nasal mucosal biopsies and immunohistochemical staining

Oral mucosa biopsies were taken from the vestibular site of the oral mucosa, without anesthesia, by using a Fokkens forceps with a cup diameter of 2.5 mm. Nasal biopsies were taken under local anesthesia by placing a cotton-wool carrier with 50–100 mg of cocaine and three drops of epinephrine (1: 100,000) under the inferior turbinate, without touching the biopsy site. Then, a mucosal biopsy sample was obtained from the lower edge of the inferior turbinate about 2 cm posterior to the edge, using a Fokkens forceps with a cup diameter of 2.5 mm. All biopsies were taken by the same investigator (S.M.R.). The biopsies were embedded in Tissue-Tek II Optimal Cutting Temperature (OCT) compound (Sakura Finetek USA Inc., Torrance, CA, USA), frozen and stored at -80°C as previously described [16].

Immunohistochemical stainings of frozen sections were performed for specific myeloid DC-markers CD1c (BDCA-1) and CD141 (BDCA-3), plasmacytoid DC-markers CD303 (BDCA-2) and CD304 (BDCA-4), and Langerhans cell-markers CD1a, and CD207 (langerin). The BDCA markers can also be expressed on other cells like B cells so we also used the morphology of the cells to identify DCs [3,4,17]. Immunohistochemical staining was performed as previously described [18]. The following mouse-anti-human mAbs were used for immunohistochemical staining by link label staining protocol: CD1a (IgG2b, 50 μg/mL, SK9, BD Pharmingen, the Netherlands), and CD207 (IgG1, 200 μg/mL, DCGM4, BD Pharmingen, the Netherlands). The following mouse-anti-human mAbs were used for immunohistochemical staining by tyramide signal amplification staining protocol: CD1c (IgG2a, 100 μg/mL, AD5-8E7, MACS, the Netherlands), CD141 (IgG1, 100 μg/mL, AD5-14H12, MACS, the Netherlands), CD303 (IgG1, 100 μg/mL, AC144, MACS, the Netherlands), and CD304 (IgG1, 100 μg/mL, AD5-17F6, MACS, the Netherlands).

#### Link label staining protocol

The mAb stainings were developed with the supersensitive alkaline phosphatase (ss-AP) method as previously described [19]. Briefly, each tissue specimen was cut into serial 5-μm-thick sections, transferred onto slides, dried, and stored at -80°C. Before staining, slides were heated to room temperature and subsequently dried and fixed in acetone for 10 min. Slides were then rinsed in phosphate-buffered saline (PBS; pH 7.4), and 10% normal goat serum (NGS) in block buffer was added for 10 min. Subsequently, slides were incubated with mouse monoclonal antibody to CD1a, or CD207 for 60 min. Sections were rinsed with PBS, incubated with goat anti-mouse immunoglobulin biotin-labeled (1:50; Biogenex) + 10% normal human serum (NHS) for 30 min, and rinsed with PBS. Slides were then incubated for 30 min with Streptavidine-AP (1:50; Biogenex) in block buffer + 10% NHS, and again rinsed with PBS. Next, slides were rinsed with Tris buffer (0.1 M, pH 8.5), and incubated with New Fuchsin substrate for 20 min. Sections were then rinsed with PBS and with distilled water. Finally sections were counterstained with Gill's hematoxyline for 4 min, rinsed in water, dried, and mounted in Vectamount. Control sections, incubated with irrelevant mAbs of the same subclass and at the same protein concentration as the specific antibody, were negative.

#### Tyramide Signal Amplification staining protocol

This staining method differed from the staining method described earlier in the inclusion of a tyramide signal amplification step (TSA; NEN Inc., Boston, MA). Briefly, tissue fixation was done in acetone for 10 min, followed by rinsing in PBS (pH 7.4), and blocking with 10% NGS in block buffer for 10 min. Subsequently, slides were incubated with mouse mAb to CD1c, CD141, CD303, or CD304 for 60 min and rinsed with PBS. Then slides were incubated with goat anti-mouse immunoglobulin biotin-labeled (1:50; Biogenex) + 10% NHS for 30 min. After rinsing in PBS, endogenous peroxidase was blocked with azide (0.3%), hydrogen peroxide (0.1%) and methanol (50%) in PBS. Slides were rinsed with PBS. This was followed by incubation with streptavidin-HRP (1:100) in block buffer + 10% NHS for 30 min, rinsing with PBS, incubation with TSA (1:40,000) in TSA-buffer for 10 min, rinsing with PBS, and incubation with AP-conjugated goat antibiotin antibody + 10% NHS (Sigma) for 30 min. This was followed by rinsing with PBS, rinsing with Tris buffer (0.1 M, pH 8.5), and incubation for 20 min with New Fuchsin substrate. The sections were rinsed in PBS and then in distilled water, counterstained with Gill's hematoxyline for 4 min, and mounted in Vectamount. Control sections, incubated with irrelevant mAbs of the same subclass and at the same protein concentration as the specific antibody, were negative.

### Analysis

Positively stained inflammatory cells, localized in epithelium and subepithelium (area 100 μm into the lamina propria along the length of the epithelial basement membrane), were counted along the basement membrane (BM). Cell numbers were expressed as positively stained cells per mm BM epithelium or per mm² of the lamina propria. Immunohistochemical staining with CD141 in oral mucosa was assessed on a 3-point scale with 0 meaning no staining, 1 some staining, and 2 extensive staining.

All data were analyzed by SPSS software. The Chi-square test was used for analysis of CD141 staining, and the Mann-Whitney U test and Kruskal-Wallis test were used for between-group analysis and all other comparisons. A *P* value less than .05 was considered significant.

## Results

### Identification of dendritic cell subsets in human oral mucosa

We identified Langerhans type, myeloid, and plasmacytoid dendritic cells in human oral mucosal biopsies taken from allergic individuals, and healthy controls (Table 1, S1 and S2 Files).

**Table 1:** Number of dendritic cell subtypes in human oral and nasal mucosa, characterized by immunohistochemical staining, in healthy and allergic subjects.

	Controls				Allergic			
	Nasal epi*	Nasal LP**	Oral Epi*	Oral LP**	Nasal epi*	Nasal LP**	Oral Epi*	Oral LP**
**mDC** CD1c	0.1 (0–1.7)	1.5 (0–15.0)	23.1 (0–78)	42.9 (5.5–114)	0.4 (0–7.9)	2.4 0–17.4)	18.8 (0–105)	43.1 (0–204)
CD141	0.4 (0–11.2)	ND	see Table 2	ND	0.5 (0–4.6)	ND	see Table 2	ND
**pDC** CD303	1.0 (0–1.2)	0.4 (0–5.0)	0.0 (0–1.2)	1.6 (0–9.7)	1.0 (0–2.1)	0.8 (0–9.0)	0.0 (0–4.0)	2.5 (0–40)
CD304	0.0 (0–2.7)	ND	0	ND	0.0 (0–7.2)	ND	0	ND
**LH** CD1a	0.9 (0–5.3)	1.3 (0–16.2)	12.3 (0–157)	6.4 (0–32)	1.1 (0–17.0)	2.6 (0–52.2)	13.1(0–210)	13.7 (0–80)
CD207	6.3 (0–20.0)	5.8 (0–18.9)	30.1(0–108)	14.7 (0–60)	8.6 (0–20.7)	4.2 (0–16.1)	48.4 (0–165)	20.5 (0–116)

* Epithelium, number of cells/mm BM, presented as median (range).

** Lamina propria, number of cells/mm^2^, presented as median (range).

**Fig 1:**
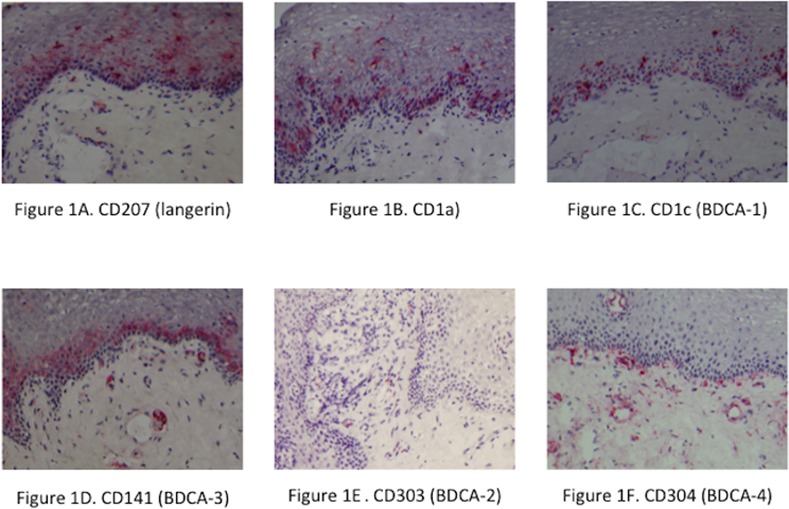
Immunohistochemical staining of a section of human oral mucosa. Staining of dendritic cell subsets was performed with specific markers: A. CD207 (langerin), B. CD1a, C. CD1c (BDCA-1), D. CD141 (BDCA-3), E. CD303 (BDCA-2), and F. CD304 (BDCA-4). Counterstained with hematoxyline. Original magnification 100x

To identify the Langerhans type cell subset we used the markers CD207 (Fig 1A) and CD1a (Fig 1B). Significant numbers of CD207+ cells where observed in both the epithelial layer and in the lamina propria (LP), but there were no significant differences between the allergic subjects (median 48.4; range 0–165 cells/mm in the epithelium, and 20.5; 0–116 cells/ mm^2^ in the LP) and healthy controls (30.1; 0–108 cells/mm in the epithelium, and 14.7; 0–60 cells/ mm^2^ in the LP). Overall, the numbers for CD207+ and CD1a+ cells were similar. Again for the CD1a+ cells in the epithelium the numbers were not different for allergic subjects (median 13.1; range 0–210 cells) or healthy controls (median 12.3; range 0–157 cells/mm). In the lamina propria however, the number of CD1a+ cells was significantly higher (p = 0.026) in the allergic subjects (median 13.7; range 0–80 cells/ mm^2^ LP) compared to the healthy controls (median 6.4; 0–32 cells/ mm^2^ LP).

To identify the myeloid DC subsets we used the markers CD1c (BDCA-1) and CD141 (BDCA-3) in combination with cell morphology. The number of CD1c cells (Fig 1C) in the allergic subjects was 18.8 cells/mm (median; range 0–105) in the epithelium and 43.1 cells/mm^2^ (median; range 0–204) in the LP, which was not significantly different from the healthy controls who had 23.1 cells/mm (median; range 0–78) in the epithelium and 42.9 cells/mm^2^ (median; range 5–114) in the LP. Immunohistochemical staining with CD141 in oral mucosa showed a confluent layer of staining, in which it was impossible to distinguish and count separate cells (Fig 1D). Therefore we decided to assess this with a categorical scale 0–2 (Table 2 and S2 File.). The amount of CD141 staining was significantly higher in allergic subjects than in healthy controls (p = 0.043).

**Table 2:** Amount of CD141+ DCs in human oral epithelium in healthy and allergic subjects.

Categories		Number of subjects		Total
		controls	allergic	
0	6	7	13
1	5	31	36
2	2	11	13
Total	13	49	62

Amount of CD141+ DCs in human oral epithelium in healthy and allergic subjects are categorized on a 3-point scale: 0 = no staining, 1 = some staining, and 2 = extensive staining.

The number of plasmacytoid DCs in the oral mucosa, as characterized by CD303 (BDCA-2) positive cells (Fig 1E), was much lower than the number of Langerhans type and myeloid DCs in both allergic and healthy individuals (Table 1). In allergic subjects the number of CD303+ DCs in the epithelium was 0.0 cells/mm (median; range 0–4.0), and in the lamina propria 2.5 cells/mm^2^ (median; range 0–40). This was not significantly different from the healthy controls, which had 0.0 cells/mm (median; range 0–1.2) in the epithelium, and 1.6 cells/mm^2^ (median; range 0–9.7) in the LP. Immunohistochemical staining with CD304 (BDCA-4; Fig 1F) was negative in the epithelium in both allergic and healthy individuals. Immunohistochemical staining with CD304 led to staining of vascular structures in the lamina propria, however no cells with dendritic cell morphology were identified.

### Oral versus nasal mucosal dendritic cells

We compared the number of the distinct DC subsets between oral and nasal mucosa in matched samples from allergic subjects and in healthy controls (Table 1). Nasal mucosal biopsy specimens revealed all subtypes of DCs, both in the epithelium and in the lamina propria, albeit at different levels. We found highest levels for CD207+ cells and the lowest levels for the pDC markers CD303 and CD304. Collectively, cell numbers in the nasal mucosa did not differ between allergic and healthy subjects. The number of Langerhans type DCs, characterized by staining with CD1a and CD207, was significantly higher in the oral mucosa. Also the number of myeloid DCs, characterized by CD1c staining, was significantly higher in the oral mucosa, compared to nasal mucosa, both in healthy and allergic subjects. Staining with CD141 in the oral mucosa showed a confluent layer of stained cells, in which it was impossible to distinguish and count separate cells. However these were evidently more than the few CD141+ cells that we found in the nasal epithelium.

In contrast, the number of plasmacytoid DCs, characterized by CD303 (BDCA-2), was comparable in the oral and nasal epithelium. The DC subset characterized by CD304 (BDCA-4) was present in low numbers in the nasal epithelium, but completely absent in the oral epithelium both in healthy and allergic subjects.

## Discussion

We demonstrated the presence of Langerhans type, myeloid, and plasmacytoid DCs, characterized by the specific DC markers CD1a and CD207, CD1c and CD141, and CD303 respectively, in human oral mucosa of allergic subjects and healthy controls. Interestingly, the use of multiple markers for pDCs and mDCs revealed a different composition of pDC and mDC subsets in human oral mucosa than has been reported for nasal mucosa and in the circulation. Moreover the oral tissue distribution for CD141 (BDCA-3) positive DCs is remarkably different than in the nasal mucosa. Furthermore, we found significantly more CD141+ myeloid DCs in epithelium and CD1a+ Langerhans cells in the lamina propria of allergic subjects compared to healthy controls. In contrast, the mDC population characterized by CD1c (BDCA-1) was similar for both allergic and healthy individuals. In the direct comparison between oral and nasal DC subtypes, we showed that the number of Langerhans type and the CD141 (BDCA-3) positive mDCs is overall much higher in oral mucosa compared to nasal mucosa. Especially the CD141 (BDCA-3) positive oral mDCs seem to form a continuous intraepithelial layer, akin the distribution of Langerhans cells in mouse dermal sheets [20,21]. It has been suggested that CD141+ DCs are the human counterpart of murine CD103+ DCs, and are the main cross-presenting DCs of human tissues [21,22].

DCs have an important role in regulation of immune responses that mainly relies on the ligation of specific receptors that initiate and modulate DC maturation. This results in the development of functionally different effector DC subsets that selectively promote different pro-inflammatory T helper subtypes or regulatory T cell responses [23]. For instance, in skin, Langerhans cells in the epithelial layer seem more adapt to detect viruses than bacteria in the environment through the presence of Toll-like receptors (TLRs) able to detect viral associated molecular patterns and the absence of TLRs able to detect bacterial associated molecular patterns. Furthermore, given that skin LCs through their poor capacity to process and present bacterial antigens are seen to induce a more tolerogenic response towards bacteria [24].

Not only in the normal antimicrobial response there is functional specialization, but also in relation to allergic disease. In respiratory mucosa, myeloid DCs play an essential role in chronic eosinophilic airway inflammation [5], while plasmacytoid DCs are important in maintaining tolerance to inhaled harmless antigen [6]. For this reason the ratio of mDCs to pDCs is often used to describe the overall immunogenic status [2,25–27]. Even though we have shown that in the nasal mucosa the mDC to pDC ratio would describe the clinical observations seen in the response to nasal allergen provocation [2], the mDC to pDC ratio is not adequate to describe the overall tolerogenic environment of the oral mucosa. As a consequence of the high number of mDCs, and the low number of pDCs in the oral mucosa the ratio of myeloid DCs over plasmacytoid DCs is much higher in the oral epithelium than in the nasal epithelium, which in contrast to clinical observations, would point to a more pro-inflammatory oral environment.

We found very low levels of plasmacytoid DCs in the oral mucosa, which suggests that pDCs overall do not play a significant role in oral tolerance, in contrast to nasal and bronchial mucosae. This led to several questions regarding other differences in response between nasal and oral mucosa with respect to the ability of DCs to induce tolerance. Given the difference in composition of DC subsets between the oral mucosa and the nasal mucosa, we consider that the oral DC subsets have different functional capacities from those in the nasal mucosa. A detailed mapping of the oral mucosa antigen-presenting cells has been performed in mice; three distinct subtypes of oral DCs were characterized: CD207+ Langerhans cells located in the mucosa itself, CD11b+CD11c+ myeloid DCs located mainly along the lamina propria, and B220+120G8+ plasmacytoid DCs in submucosal tissues [7,28]. Data on DC phenotype within human oral mucosa are limited. The Langerhans type (CD207+) cells were thought to represent the predominating DC population in human oral mucosa [9,10]. The presence of myeloid DCs, characterized by CD1a, in human oral mucosa has also been shown [11], but plasmacytoid DCs, characterized by CD123+, could previously not be detected in atopic or nonatopic individuals [12].

Our survey of the oral mucosa revealed abundant numbers of CD141+ myeloid dendritic cells, and CD207 and CD1a positive Langerhans cells. Given the postulated ability of (skin) Langerhans cells to induce tolerogenic responses towards bacteria these high numbers could be in line with the notion of the oral tolerogenic environment. Possibly tolerogenic response towards bacteria could be extended towards the uptake and processing of allergens as well, based on similarities in molecular patterns. DCs in the human oral mucosa express the high-affinity receptor for IgE (FcεRI) in high amounts [9], in which context they differ from DCs in the nasal mucosa. Interestingly this is not only seen in atopic individuals, but also in non-atopic individuals [29]. Fc mediated uptake could therefore be involved in the direct uptake of allergens by DCs in the oral mucosa [30]. The direct contact between DCs and T cells observed within the oral mucosa [29] and the regulatory T cell inducing capacity of Langerhans cells could therefore be seen to contribute to a local immunosuppressive environment.

Another potentially important observation in our study is the dense intraepithelial network of CD141+ DCs in the oral mucosa of allergic subjects. As myeloid DCs are thought to be immunogenic the implication is that the pro-inflammatory immune response to aero-allergens could either be driven from the oral mucosa in allergic subjects, or represents a lack of tolerogenic properties in the oral mucosa in allergic subjects. This aspect is specific for oral mucosa, as in a previous study we did not find a significant difference in the number of CD141+ DCs in the nasal mucosa between allergic subjects and healthy controls [2]. Moreover in a mouse model of bronchial asthma, CD141+ DCs were shown to be tolerogenic. They expressed lower levels of maturation markers, secreted more anti-inflammatory cytokine IL-10, and adoptive transfer of CD141+ DCs caused a reduction in BALF levels of eosinophils, IL-5, IgE, and total protein [31].

In conclusion, we have shown the abundance of Langerhans cells and CD141+ myeloid dendritic cells in the oral mucosa of allergic and healthy individuals, and our work is the first report of CD303+ plasmacytoid dendritic cells in oral mucosa. The direct comparison between the distribution of these DC subtypes between oral and nasal mucosa in the same individuals revealed potentially important differences. These differences suggest that the suppression of immune responses could be regulated in distinct fashions at both mucosal surfaces. A better understanding of these mechanisms could lead to optimization of sublingual immunotherapy in particular.

## Supporting Information

S1 File Nasal DC cell counts. (XLSX)

S2 File Oral DC cell counts. (XLSX)
